# METTL3 regulates N6-methyladenosine modification of ANGPTL3 mRNA and potentiates malignant progression of stomach adenocarcinoma

**DOI:** 10.1186/s12876-023-02844-x

**Published:** 2023-06-21

**Authors:** Zhijin Zhang, Jun Fu, Yuhao Zhang, Xianju Qin, Yuexia Wang, Chungen Xing

**Affiliations:** 1 Department of General Surgery, the Second Affiliated Hospital of Soochow University, No. 1055, Sanxiang Road, Suzhou, 215004 Jiangsu China; 2 Department of General Surgery, Shanghai Eighth People Hospital, Shanghai, 200235 China

**Keywords:** Stomach adenocarcinoma, METTL3, N6-methyladenosine, ANGPTL3

## Abstract

**Background:**

N6-methyladenosine (m6A) is associated with mammalian mRNA biogenesis, decay, translation and metabolism, and also contributes greatly to gastrointestinal tumor formation and development. Therefore, the specific mechanisms and signaling pathways mediated by methyltransferase-like 3 (METTL3), which catalyzes the formation of m6A chemical labeling in stomach adenocarcinoma (STAD), are still worth exploring.

**Methods:**

Quantitative real-time PCR (qRT-PCR) was constructed to detect the expression of METTL3 in gastric cancer cell lines and patient tissues. The biological function of METTL3 was investigated in vitro/in vivo by Cell Counting Kit-8, colony formation assay, Transwell assay and nude mouse tumorigenesis assay. Based on the LinkedOmics database, the genes co-expressed with METTL3 in the TCGA STAD cohort were analyzed to clarify the downstream targets of METTL3. Methylated RNA immunoprecipitation-qPCR (MeRIP-qPCR) and RNA stability analysis were employed to explore the mechanism of METTL3 in gastric cancer progression.

**Results:**

We analyzed TCGA data and found that METTL3 was frequently elevated in STAD, and demonstrated that METTL3 was present at high levels in clinical STAD tissues and cells. High METTL3 expression was more likely to have advanced TNM tumors and distant metastasis. On the other hand, METTL3 silencing effectively impeded the higher oncogenic capacity of AGS and HGC27 cells in vivo and in vitro, as reflected by slowed cell growth and diminished migration and invasion capacities. Continued mining of the TCGA dataset identified the co-expression of angiopoietin-like 3 (ANGPTL3) and METTL3 in STAD. Lower level of ANGPTL3 was related to increased level of METTL3 in STAD samples and shorter survival times in STAD patients. ANGPTL3 enrichment limited the growth and metastasis of STAD cells. Besides, ANGPTL3 mRNA levels could be decreased by METTL3-dominated m6A modifications, a result derived from a combination of MeRIP-qPCR and RNA half-life experiments. Importantly, the inhibitory effect of METTL3 silencing on cancer could be reversed to some extent by ANGPTL3 inhibition.

**Conclusions:**

Overall, our findings suggested that METTL3 functioned an oncogenic role in STAD by reducing ANGPTL3 expression in an m6A-dependent manner. The discovery of the METTL3-ANGPTL3 axis and its effect on STAD tumor growth will contribute to further studies on the mechanisms of gastric adenocarcinoma development.

**Supplementary Information:**

The online version contains supplementary material available at 10.1186/s12876-023-02844-x.

## Introduction

Gastric cancer (GC) is a highly fatal disease, with an estimated increase of at least 1 million new cases and 760,000 deaths worldwide in 2020 [1]. Among all gastric cancer cases, the majority of patients were diagnosed as stomach adenocarcinoma (STAD) arising from malignant change of gastric gland cells, accounting for 90–95% of total cases [2]. The causative factors of STAD are commonly attributed to environmental stimuli (mainly H. pylori infection) and genetic susceptibility [3]. Despite the availability of multidisciplinary combination therapy including surgery and chemotherapy for STAD patients in the past decades, clinical outcomes remain suboptimal and median survival time has not been effectively prolonged [4]. Heterogeneity at the molecular level is one of the main reasons affecting STAD treatment outcomes. Therefore, it is essential to explore and discover new promising biomarkers to identify specific stages of tumors for personalized treatment of patients.

Tumor growth and metastasis involve the dysregulation of key genes [5]. Moreover, reversible N6-methyladenosine (m^6^A) modification is a novel form emerging in the regulation of gene expression and the most prominent and widespread RNA internal chemical marker in higher eukaryotes, present in more than a quarter of messenger RNAs (mRNAs) [6, 7]. Thus, there is a close functional relationship between altered intracellular m^6^A levels and abnormal proliferation status of cancer cells [8]. Yang and colleagues showed that methyltransferase-like 3 (METTL3) mediated the raise in m^6^A levels in MYC, which in turn induced enhanced growth and invasion of GC cells [9]. The multiprotein “writer” complex (WTAP-METTL3-METTL14), “readers” (YTHDF1, YTHDF2, YTHDF3) and “erasers " (ALKBH5 and FTO) can dynamically control the deposition, recognition or erasure of m^6^A at specific mRNA loci [10, 11]. In other words, dynamic reversible m6A modifications are orchestrated by writers and erasers, while the function of m6A in mRNA metabolism is performed by readers [12]. M6A sites are located in the DRACH (D = A/G/U, R = A/G, H = A/C/U) consensus motifs and are enriched in the vicinity of the 3’-UTRs and stop codons of mRNA [12]. M6A “readers” selectively bind methylated mRNAs and control RNA fate in a methylation-dependent manner, including mRNA stability, mRNA decay, mRNA translation, etc. Methyltransferase-like 3 (METTL3) can determine the fate of target RNAs, and its ablation or overexpression frequently leads to altered m6A levels in a variety of malignancies, which subsequently recruit m6A-binding proteins to modify mRNA decay and translation, ultimately inhibiting or promoting disease progression [13]. Elevated METTL3 strengthened the m6A levels of APC mRNA in esophageal squamous cell carcinoma and subsequently recruited YTHDF to degrade APC mRNA, ultimately activating cancer cell proliferation and aerobic glycolysis [14]. In addition, METTL3-mediated m^6^A modifications were translated into pro-tumorigenic signals in colorectal cancer [15]. And METTL3 restricted YPEL5 expression in an m6A-YTHDF2-dependent manner also became an important cause of colorectal cancer growth and metastasis [16]. In gastric cancer, aberrant METTL3 has long been recognized as a marker of poor patient prognosis, and enhanced METTL3 up-modulated m^6^A modification of ZMYM1 mRNA [17], DEK mRNA [18] or HDGF mRNA [19] to induce tumor growth and distant metastasis. However, cancer development involves multi-gene and multi-step alterations, and the relevant expression mechanisms of m^6^A-regulated genes in gastric cancer still need to be infused with new insights.

Overall, we designed in vivo and in vitro experimental protocols in STAD to confirm that METTL3 was a key promoter of tumor growth and metastasis. Most interestingly, using bioinformatics analysis, we found that METTL3 boosted m^6^A modification of angiopoietin-like 3 (ANGPTL3) mRNA in STAD. METTL3 limited the stability and abundance of ANGPTL3 and enhanced several key biological behaviors (proliferation, migration and invasion) of cancer cells. Our work refined the understanding of METTL3-involved m^6^A signaling and injected new insights into the field of stomach adenocarcinoma research.

## Materials and methods

### STAD tissue samples and cell culture

A total of 62 pairs of tumor samples (STAD specimens and paired adjacent normal tissue specimens) resected during surgery were collected from Shanghai Eighth People Hospital for this study. Patients who had been systematically treated and those with other diseases were not allowed to be included in this study. The study protocol for the use and collection of human tissues was reviewed and approved by our ethics committee. All participants signed a written consent form before being subjected to the study. All experiments were conducted in strict compliance with relevant guidelines and regulations and in accordance with the *Declaration of Helsinki*. Table 1 listed the relationship between METTL3 expression and clinical-histopathological characteristics of STAD patients.

**Table 1:** The correlation between METTL3 expression and clinical-histopathological characteristics in STAD patients

Clinical Features	No.	METTL3 expression		*P* value
		Low	High	
Total	62	31	31	
Gender				0.1684
Male	43	24	19	
Female	19	7	12	
Age				0.7957
< 60	37	19	18	
≥ 60	25	12	13	
Smoking				0.4373
Yes	25	14	11	
No	37	17	20	
Tumor location				0.8707
Upper	15	7	8	
Middle	23	11	12	
Lower	24	13	11	
Size of tumor(cm)				0.4303
< 5	39	21	18	
≥ 5	23	10	13	
TNM stage				0.0020*
I + II	26	19	7	
III + IV	36	12	24	
Distant metastasis				0.0169*
M0	58	37	21	
M1	14	4	10	
*H.pylori*				0.5758
Positive	18	10	8	
Negative	44	21	23	

*P* value was calculated with chi-square test and *P < 0.05 was recognized as a significant difference

The human gastric mucosal cell line GES1 and two human gastric cancer cell lines (AGS and HGC27) involved in this study could be obtained from the Cell Bank of the Chinese Academy of Sciences (Shanghai, China). GES1, AGS and HGC27 could all be grown in commercially available medium (RPMI 1640, Gibco, USA), according to the manufacturer’s recommendations. Notably, penicillin/streptomycin solution (1%, 100 U/mL, HyClone, UT) and 10% FBS (Gibco, USA) need to be routinely supplemented into RPMI 1640 basal medium. Finally, the culture flasks were placed in a humidified incubator (culture environment: 37 °C, 5% CO_2_) to maintain cell growth.

### Bioinformatics Analysis

The expression profiles of 19 m^6^A regulators in STAD were extracted from The Cancer Genome Atlas (TCGA) dataset. Genes co-expressed with METTL3 in the TCGA STAD cohort were evaluated based on the LinkedOmics database, and data were visualized using volcano plots. The online SRAMP prediction tool was manipulated to predict the potential m^6^A modification sites present in ANGPTL3 sequences.

### Cell transfection

Small interfering RNAs (si-METTL3 1#, si-METTL3 2#, si-ANGPTL3 1# and si-ANGPTL3 2#) targeting specific regions of METTL3 and ANGPTL3 were designed and synthesized by Obio Technology (Shanghai, China). The lentiviral vector mediating low expression of METTL3 (sh-METTL3) and its control lentiviral vector (sh-NC) were ordered from Anhui General Biological Company (China). Stable low expression of METTL3 in AGS cells was achieved by lentiviral transfection and screening (puromycin, 2 µg/ml). The CDS region of ANGPTL3 was amplified and inserted into pcDNA (Invitrogen, USA) vector to construct ANGPTL3 overexpression plasmid. The vector for knocking down METTL3 and over-expressing ANGPTL3 and siRNA targeting the specific genes were transfected with HGC27 and AGS cells using Lipofectamine 3000 (Invitrogen, USA), according to the manufacturer’s instructions.

### In vitro cell viability assay

Two STAD cell lines (AGS and HGC27) were evaluated for proliferative capacity via the Cell Counting Kit-8 (CCK-8, RiboBio, China) method. Cell suspensions of STAD cells transfected with over-expressed vectors or siRNAs for the indicated genes were prepared and subsequently injected into 96-well plates (1 × 10^3^ cells/well, 100 µL). At specific time points (0d, 2d, 4d and 6d), 10 µL of CCK-8 reaction solution (ApexBio, USA) was added to the cell cultures for continued maintenance for 2 h. Finally, the optical density (OD) values of each well were recorded at 450 nm using a multifunctional zymograph (Tecan M200 PRO, Belgium).

### RNA half-life measurement

AGS cells and HGC27 cells were processed with siRNA against METTL3. 48 h later, the indicated cells were mixed with actinomycin D (5 µg/ml, AAT Bioquest) to block the synthesis of new RNA in the cells. Next, qRT-PCR was performed at the indicated time points (0 h, 8 h, 16 h, 24 h) for stability analysis of ANGPTL3 mRNA.

### Measurement of colony formation

Treated AGS or HGC27 cells (1 × 10^3^) were spread in six-well plates (2 ml RPMI 1640) to examine their colony-forming ability. After 14-day of continuous incubation, the target cells were fixed with methanol (Merck) and stained with crystal violet (0.1%, 30 min). Finally, the staining solution was discarded, the colonies were washed, and the colonies were imaged and counted under a microscope (Olympus).

### Transwell experiment

Twelve-well 8 μm Transwell chambers (BD Biosciences) were employed to evaluate migration and invasion of AGS or HGC27 cells. The transfected STAD cells were counted and prepared into cell suspensions at a density of 2 × 10^5^ cells/mL. For migration assay, 200 µL of cell solution was dispensed into the top compartment, while 800 µL of chemical induction agent (RPMI1640 medium + 20% FBS) was added to the basolateral compartment. After being maintained in the incubator for 24 h, the top compartment was removed and immersed in methanol for 20 min to fix the cells, followed by 0.1% crystal violet staining for 30 min (room temperature). Residual cells on the upper surface of the upper chamber were manually wiped off with a wet cotton swab. At least 4 random fields of view were selected for photographing and counting under a 200×Olympus inverted microscope. For the invasion assay, 50 µL of Matrigel solution needs to be pre-coated in the upper chamber of the Transwell and incubated to solidify. Other steps are similar to the migration assay. For the invasion assay, 50 µL of matrix gel solution needed to be spread on the Transwell upper chamber and incubated to solidify, and other operational steps were similar to migration experiments.

### Western blotting analysis

AGS and HGC27 cells were lysed in RIPA lysate (P0013C, Beyotime, Shanghai, China) and whole proteins were extracted, which need to be supplemented with 1% protease inhibitor (Roche, USA) mixture. Protein concentrations were estimated using the BCA Protein Assay Kit (P0010S, Beyotime, Shanghai, China). Equal amounts of denatured protein samples (30 µg) were resolved on 10-12% SDS-PAGE and subsequently transferred to PVDF (Millipore, USA) membranes. The membranes were closed with 5% skim milk for 2 h and incubated overnight at 4 °C with diluted primary antibodies specific for METTL3 (1:500, Abcam), ANGPTL3 (1:1,000, Abcam), and GAPDH (1:1,000, Abcam). The membranes were continued to be incubated with secondary antibodies for 1.5 h before visualization of protein blot images using the ECL kit (Millipore, USA).

### qRT-PCR

Total RNA was isolated from STAD tissue specimens, AGS and HGC27 cells using an RNA extraction kit (TRIzol, Invitrogen, USA). The purity and concentration of the total RNA obtained were determined, and then the generation of cDNA templates was initiated using the HiScript II kit (Vazyme, China). Next, the PCR kit (SYBR® Premix Ex Taq™, Takara) and Light Cycler 480 II system (Roche, China) were employed to complete the remaining qRT-PCR steps. Levels of METTL3 and ANGPTL3 were fully normalized to β-Actin. Relative expression levels of target genes were calculated by 2 ^–∆∆Ct^ method. The primer sequences of the specific genes used were detailed:

METTL3-F: 5’-AAGCTGCACTTCAGACGAAT-3’

METTL3-R: 5’-GGAATCACCTCCGACACTC-3’

ANGPTL3-F: 5’-CTTCAATGAAACGTGGGAGAACT-3’

ANGPTL3-R: 5’-GTAATCGCAACTAGATGTAGCGT-3’.

β-Actin-F: 5’-TGAGAGGGAAATCGTGCGTGAC-3’.

β-Actin-R: 5’-AAGAAGGAAGGCTGGAAAAGAG-3’.

### MeRIP-qPCR

The Magna MeRIP™ m6A kit (Millipore, USA) was utilized to determine the enrichment level of m^6^A on ANGPTL3 sequences. Briefly, total RNA was obtained from AGS and HGC27 cells transfected with si-METTL3, using cells transfected with si-NC as control. Subsequently, mRNA Purification Kit (Invitrogen) and fragmentation buffer were used separately to purify and fragment total RNA (50 µg) in each sample. Anti-m^6^A antibody (Abcam) or anti-IgG was co-immunoprecipitated for 2 h at 4 °C with the obtained purified mRNA in IPP buffer, which contains pretreated protein A/G magnetic beads (Thermo Scientific). Finally, the corresponding m^6^A enrichment levels in the co-precipitated RNA samples were analyzed and calculated on a 7500 Fast RT-PCR instrument (Applied Biosystems).

### Animal Research

Twelve 8-week-old female BALB/c nude mice (weight 18–20 g, SLAC Laboratory Animal Co., Ltd., Shanghai, China) were operated for the in vivo xenograft tumor experiments. Animals use and experimental procedures were in strict accordance with institutional guidelines and were approved by the Second Affiliated Hospital of Soochow University Ethics Committee. For in vivo tumor formation, AGS cells (5 × 10^7^) stably infected with sh-METTL3 lentivirus or sh-NC control cells were injected subcutaneously into nude mice (n = 3). The volume and size of the tumors were observed and recorded every three days from the third day after inoculation, calculated as 0.5×(length×width^2^). After three weeks, all nude mice were executed and the tumor tissues were immediately excised for photographing and weighing.

### Statistical analysis

The experiments used were performed at least three times independently and the data obtained are reported as mean ± SD. Statistical differences between two or more consecutive data sets were compared using Student’s *t*-test and one-way analysis of variance (ANOVA). Pearson correlation analysis was performed to demonstrate the correlation between METTL3 and ANGPTL3 expression. Graphs were plotted using GraphPad Prism 8.0 software. *P*-values less than 0.05 were considered statistically significant.

## Results

### High expression of METTL3 in metastatic stomach adenocarcinoma induces cancer progression

**Fig. 1:**
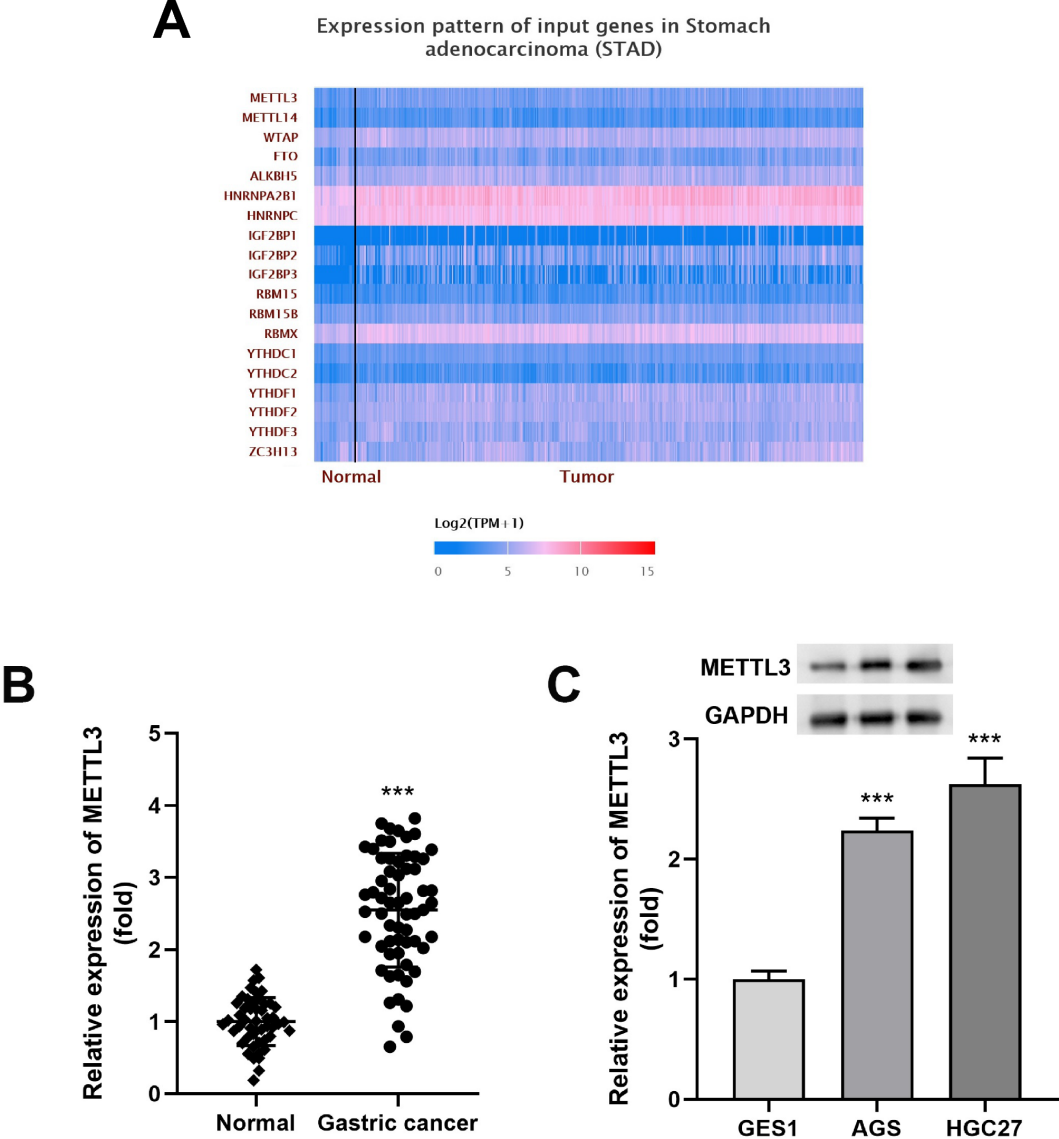
METTL3 was present in a high expression pattern in stomach adenocarcinoma. **(A)** Expression levels of m^6^A regulators in STAD were analyzed in the TCGA database. **(B)** Expression of METTL3 in 62 clinical STAD tumor tissues (Tumor) and paired normal tissues (Normal) was collected by qRT-PCR assay. **(C)** Differences in METTL3 mRNA and protein expression levels in human gastric mucosal cells (GES1) and representative STAD cells (AGS and HGC27). The membrane for Western blotting was cropped according to the molecular weight of the protein prior to incubation with primary antibodies. ***P < 0.001

To assess the mRNA expression profile of m^6^A-related genes in human STAD, we analyzed the TCGA database, and the heat map evidenced that several m^6^A-related genes were severely dysregulated in STAD tissues. Among them, METTL3, WTAP, YTHDF1, YTHDF2, YTHDF3 and HNRNPA2B1 showed a consistent upregulation trend in the dataset (Fig. 1A). In human pathophysiology, METTL3 was considered an essential factor that functioned a crucial role in multiple stages of the RNA life cycle [20, 21], and its upregulation could influence m^6^A modifications in mRNAs of key target genes to stimulate tumor formation. Moreover, unlike other m6A regulators, METTL3 was widely participated as an oncogene in different aspects of gastric cancer progression, including cell growth, survival and metastasis [22]. Importantly, the potential mechanisms of METTL3 were complex, involving multiple molecules and pathways in gastrointestinal cancer, but its function and clinical outcomes in STAD required further investigation. In the current study, we confirmed a consistent rise of METTL3 in 62 previously obtained STAD tumor tissues, but this was relative to paracancerous normal samples (Fig. 1B and Additional file 1: Figure S1). Based on the obtained results, in order to evaluate the clinical value of METTL3, we analyzed the relationship between METTL3 expression levels and clinical-histopathological characteristics of STAD patients. First, STAD patients were divided into METTL3 high expression group (n = 31) and METTL3 low expression group (n = 31) according to the median expression of METTL3. Subsequently, the clinical manifestations of the subject patients were compared. As shown in Table 1, patients in the METTL3 high expression group were more likely to present with higher tumor stage (III + IV, *P* = 0.0020) and distant metastasis (*P* = 0.0169). However, no correlation was found between circ_002136 levels and other clinicopathological factors, such as age (*P* = 0.7957), gender (*P* = 0.1684), smoking (*P* = 0.4373), tumor location (*P* = 0.8707), tumor size (*P* = 0.4303) and H.pylori (*P* = 0.5758). Furthermore, elevated expression levels of METTL3 were also found in invasive STAD cell lines (AGS, HGC27), using GES1 cells as a reference (Fig. 1C). In agreement with the above findings, western blot clarified an increase of METTL3 expression in AGS and HGC27 (Fig. 1C). These data suggested that enhanced METTL3 might exert a particular role in STAD and could be a candidate molecule for abnormal m^6^A modifications in specific diseases.

### Lower METTL3 level hinders the malignant behaviors of stomach adenocarcinoma cells

**Fig. 2:**
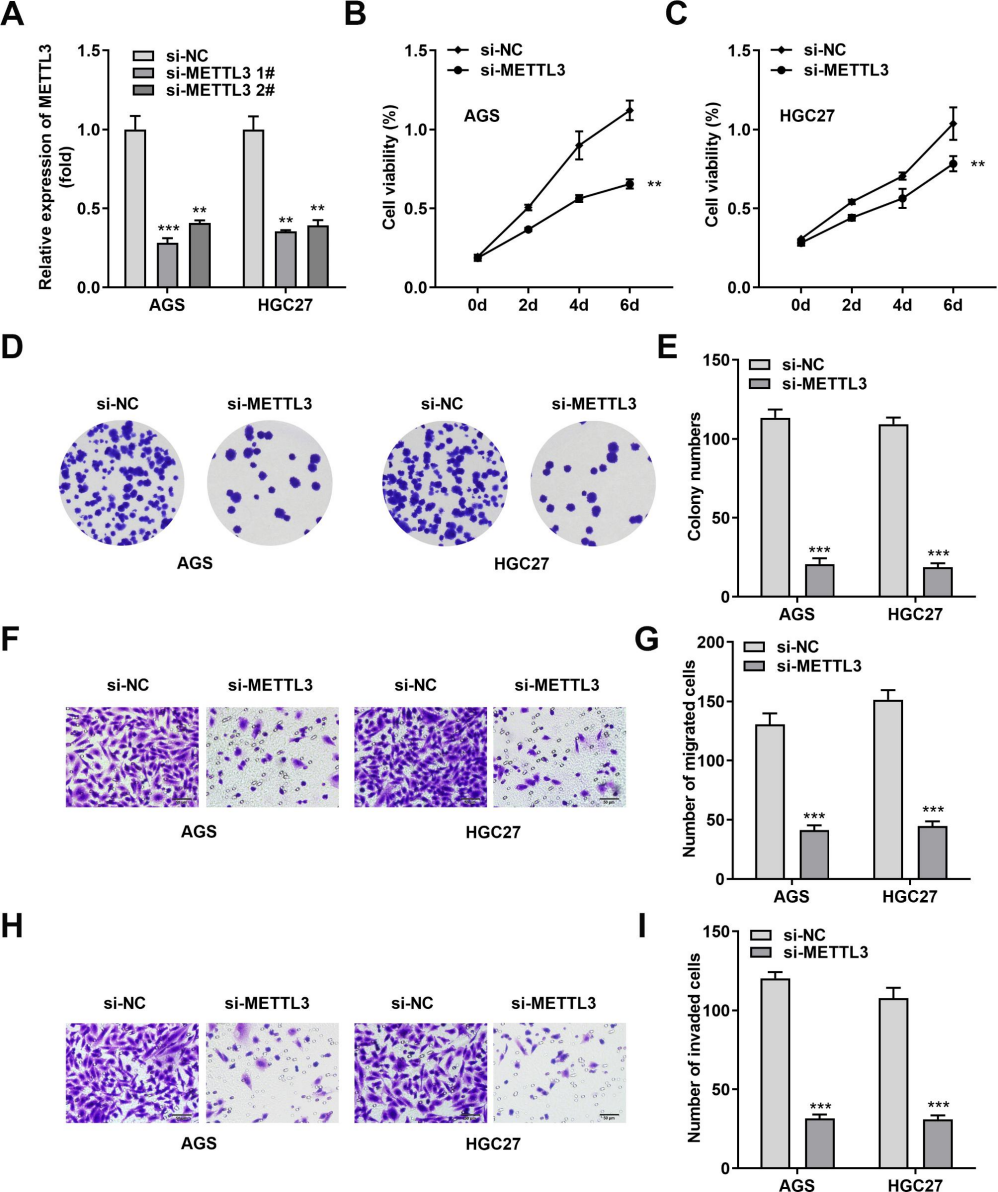
STAD cells proliferation, migration and invasion were limited by decreased METTL3 level. **(A)** The expression of METTL3 in AGS and HGC27 was successfully depleted by si-METTL3 1# and si-METTL3 2#. Among them, si-METTL3 1# presented stronger knock-down efficiency. **(B, C)** CCK-8 assay was utilized to obtain cell viability data. **(D, E)** Colony formation assay was developed to obtain changes in cell proliferation capacity. The number of AGS and HGC27 cell clones was calculated by the received microscopic images. **(F-I)** Transwell experiments illustrated that METTL3 knockdown restrained the migration and invasion of AGS and HGC27 cells. The number of AGS and HGC27 cells migration and invasion was calculated from the collected images. **P < 0.01, ***P < 0.001

To explore the oncogenic properties of METTL3 in tumors, we successfully and specifically interfered with METTL3 expression using si-METTL3 1# and si-METTL3 2# in two STAD cells (AGS, HGC27) (Fig. 2A). As expected, once the intracellular METTL3 levels were successfully knocked down, the viability of AGS and HGC27 cells was significantly decreased (Fig. 2B C). In addition, AGS and HGC27 cells also showed the same trend of significantly diminished colony formation capacity (Fig. 2D and E). The data of Transwell assay indicated that the number of migrating and invading AGS and HGC27 cells decreased after si-METTL3 treatment (Fig. 2F and I). In conclusion, the attenuation of METTL3 could weaken the malignant biological behaviors of the STAD cells.

### ANGPTL3 is negatively associated with METTL3 in stomach adenocarcinoma

**Fig. 3:**
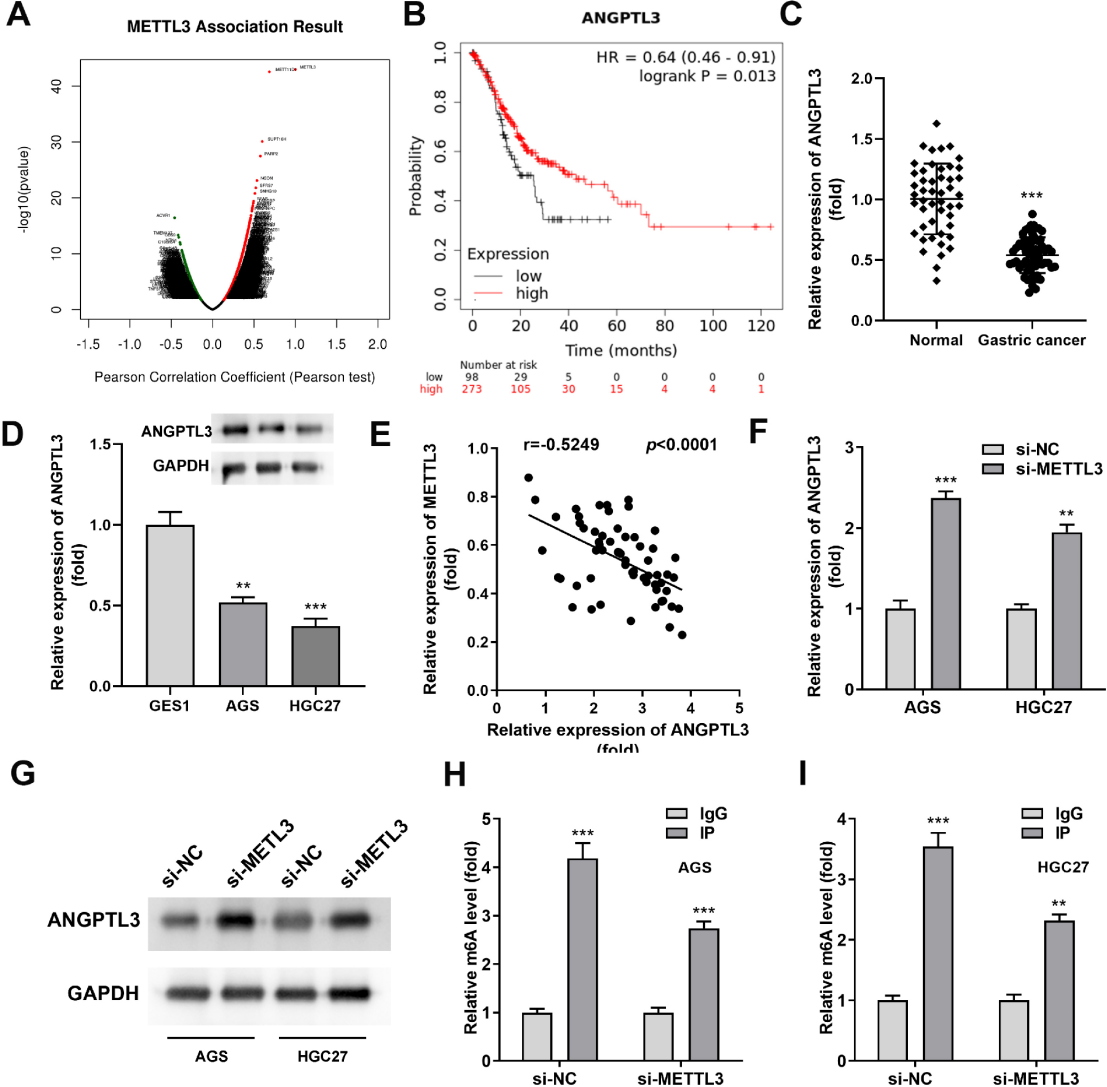
METTL3 negatively regulated ANGPTL3 levels in stomach adenocarcinoma. **(A)** Volcano plot was plotted to show the genes highly associated with METTL3 in the TCGA STAD dataset. **(B)** Kaplan-Meier survival curves were employed to demonstrate the difference in overall survival time of STAD patients in two subgroups (ANGPTL3 high and ANGPTL3 low expression) in the TCGA database. **(C)** ANGPTL3 was significantly reduced in 62 primary STAD tissues, using normal tissues adjacent to the cancer as controls. **(D)** Reduced mRNA and protein levels of ANGPTL3 were found in two STAD cell lines (AGS and HGC27). **(E)** The correlation between METTL3 and ANGPTL3 was received based on qRT-PCR data. **(F)** Relative levels of ANGPTL3 mRNA were detected in AGS and HGC27 cells transfected with si-METTL3. **(G)** Western blot analysis was used to detect the effect of METTL3 silencing on ANGPTL3 protein expression in AGS and HGC27 cells. The membrane for Western blotting was cropped according to the molecular weight of the protein prior to incubation with primary antibodies. **(H, I)** MeRIP-PCR was applied to calculate the level of m^6^A modification of ANGPTL3 after METTL3 silencing. **P < 0.01, ***P < 0.001

To continue exploring the potential implications of METTL3 in STAD and to clarify its downstream targets in STAD, we investigated co-expressed genes in STAD associated to METTL3 expression in the LinkedOmics database. The volcano plot presented (Fig. 3A) that METTL3 was positively associated with 2081 genes (red dots) and negatively associated with 1532 genes (green dots). Determining the threshold as cor > 0.2 and *P* < 0.05, ANGPTL3 revealed strong negative correlation with METTL3. To clarify that dysregulation of ANGPTL3 was a critical event in STAD, we performed further survival analysis on 273 high ANGPTL3 expressing STAD samples and 98 low expressing samples in the TCGA dataset. Kaplan-Meier curves manifested that high ANGPTL3 expressing patients exhibited longer overall survival than low ANGPTL3 expressing patients (Fig. 3B, P = 0.013). Moreover, the levels of ANGPTL3 in representative STAD tissue samples were also significantly lower than that in para-cancerous tissues (Fig. 3C). Furthermore, the reduced ANGPTL3 mRNA and protein levels were also consistently found in conventional gastric cancer cell lines (AGS and HGC27) (Fig. 3D). Notably, ANGPTL3 displayed a significant negative correlation with METTL3 in a cohort of 62 pairs of gastric cancer tumors and paraneoplastic normal tissues obtained from our institution (Fig. 3E, P < 0.0001). Based on the obtained data, we speculated that the dysregulation of ANGPTL3 levels in STAD might be regulated by METTL3. As shown in Fig. 3F, the introduction of si-METTL3 in cells induced an increase in ANGPTL3 levels. In addition, in METTL3 knockdown cells, ANGPTL3 protein expression levels also showed changes consistent with mRNA levels (Fig. 3G). The m^6^A levels of ANGPTL3 mRNA in AGS and HGC27 cells also revealed a consistent upward trend (Fig. 3H and I). The above data demonstrated that METTL3 might negatively regulate ANGPTL3 levels in invasive gastric cancer cells by means of the m^6^A pathway.

### The tumor suppressor effect of METTL3 silencing can be relieved to some extent by ANGPTL3 inhibition

**Fig. 4:**
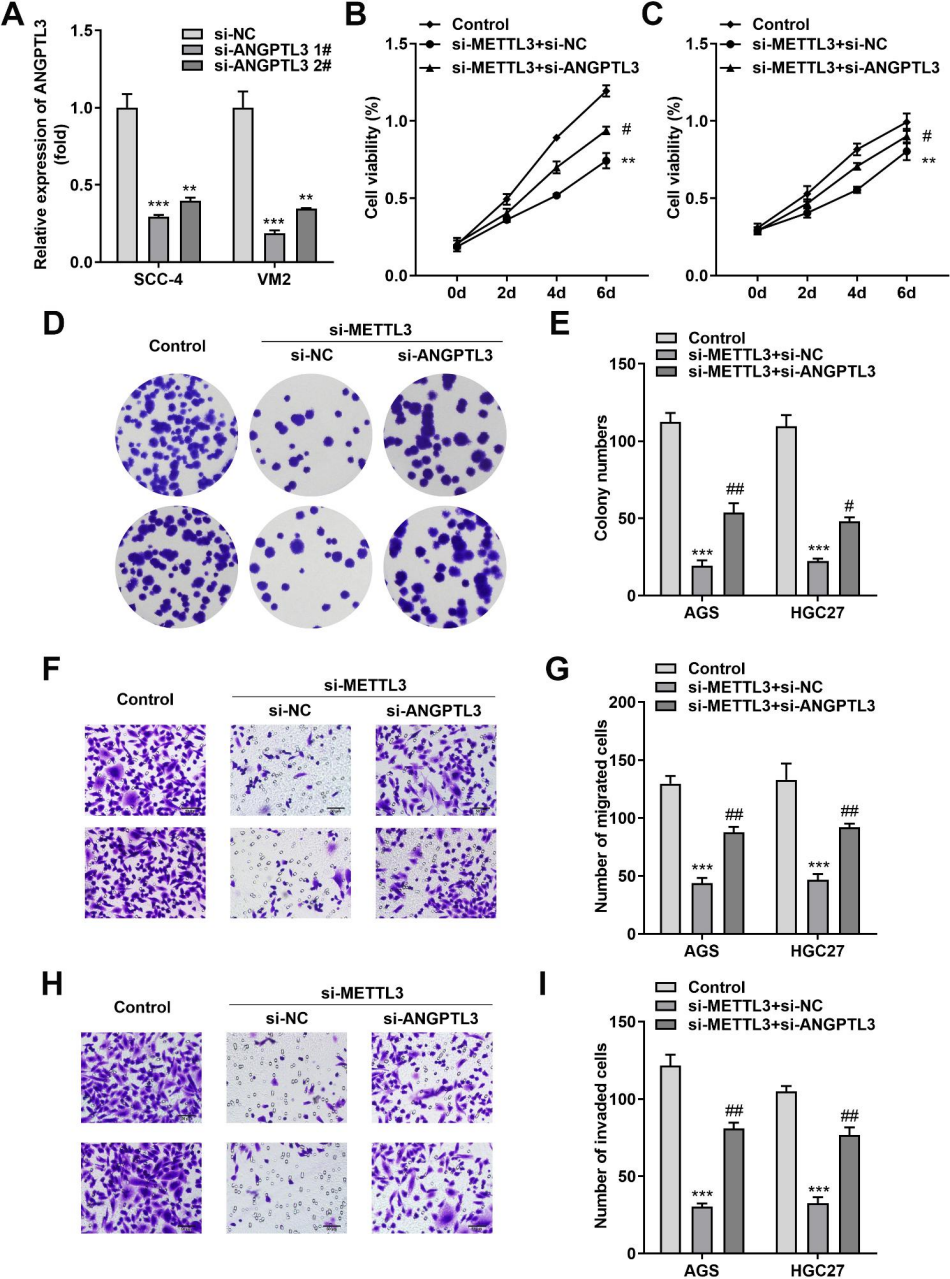
ANGPTL3 silencing rescued si-METTL3-impeded proliferation, migration and invasion of STAD cells. AGS and HGC27 cells were transfected with Control, si-METTL3 + si-NC and si-METTL3 + si-ANGPTL3, respectively. **(A)** The knockdown efficiency of si-ANGPTL3 1# and si-ANGPTL3 2# was verified using qRT-PCR. **(B, C)** CCK-8 assay was managed to detect the effect of ANGPTL3 silencing on METTL3-knockdown AGS and HGC27 cells. **(D, E)** The joint effect of si-ANGPTL3 and si-METTL3 on STAD cell proliferation was assessed by colony formation assay. Microscopy captured images (D, left) and counted the number of colonies (E, right). **(F-I)** Altered migratory and invasive capacity of si-METTL3 + si-NC and si-METTL3 + si-ANGPTL3-transfected AGS and HGC27 cells were examined using the Transwell system. The number of migrating and invading AGS and HGC27 cells was calculated from the obtained images. **P < 0.01, ***P < 0.001, ^#^*P* < 0.05, ^##^*P* < 0.01

To estimate whether ANGPTL3 was relevant to STAD cell growth and invasion, a gain-of-function study was performed. qRT-PCR results revealed the overexpression efficiency of the two cells after transfection (Additional file 1: Figure S2A). We investigated the effect of ANGPTL3 on cell growth, and CCK-8 results showed that strengthened ANGPTL3 hindered the growth of AGS and HGC27 (Additional file 1: Figures S2B and S2C). In addition, colony formation analysis demonstrated that ANGPTL3 overexpression reduced the number of clones in AGS and HGC27 cells (Additional file 1: Figures S2D and S2E). Similarly, the migratory and invasive abilities of AGS and HGC27 cells were significantly inhibited upon ANGPTL3 overexpression (Additional file 1: Figures S2F-S2H). Next, we continued to focus on the relationship between ANGPTL3 and the biological properties of STAD cells and operated two siRNAs (si-ANGPTL3 1# and si-ANGPTL3 2#) to specifically knock down ANGPTL3 expression in STAD cells. As shown in Fig. 4A, the introduction of si-ANGPTL3 1# and si-ANGPTL3 2# successfully sharply reduced the intracellular ANGPTL3 levels, with si-ANGPTL3 1# being more efficient in knocking down. Next, we transfected si-METTL3 alone or co-transfected with si-ANGPTL3 into AGS and HGC27 cells for exploring whether ANGPTL3 was involved in cell growth and metastasis impeded by si-METTL3. CCK-8 assays presented that STAD cells viability was weakened in response to attenuation of METTL3 levels, while ANGPTL3 small interfering RNA partially increased the viability of tumor cells depleted by METTL3 silencing (Fig. 4B C). In addition, colony formation assays determined the effect of si-ANGPTL3 on the colony formation of AGS and HGC27 cells. After 14 days of culture, the number of colonies in the si-METTL3 + si-ANGPTL3 group was significantly more than that in the si-METTL3 + si-NC group, while diminished colony formation capacity was characteristic of the si-METTL3 + si-NC group (Fig. 4D and E). Finally, the Transwell assay further confirmed that si-METTL3 induced a downregulation of STAD cell migration and invasion abilities, and this effect could be overridden by ANGPTL3 silencing (Fig. 4F and I). In conclusion, these data suggested that ANGPTL3 siRNA could deplete the antitumor effects of METTL3 knockdown and synergistically activate the production of an “aggressive” cell phenotype.

### METTL3 represses ANGPTL3 mRNA stabilization in an m^6^A-dependent pathway

**Fig. 5:**
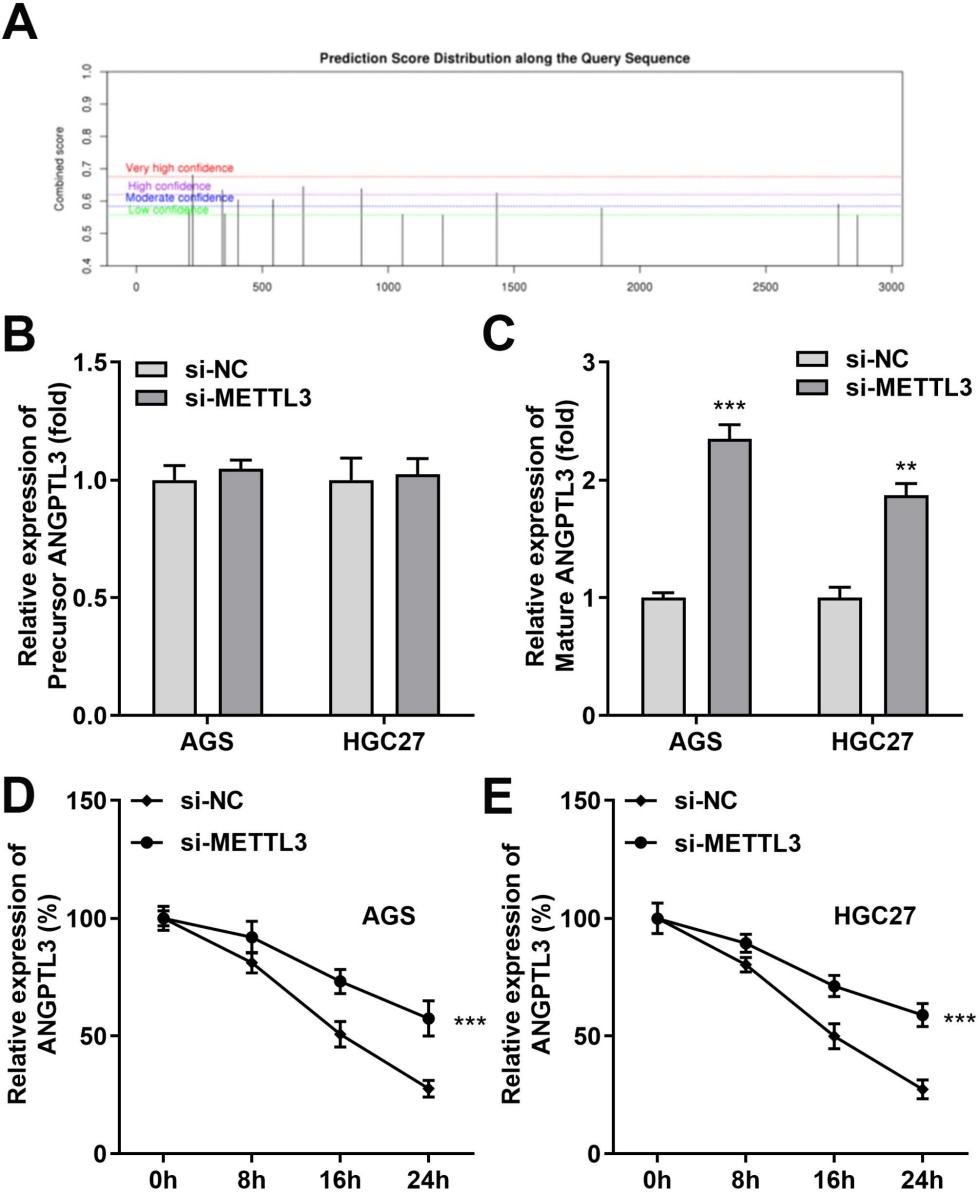
METTL3 silencing promoted the stability of ANGPTL3 mRNA. **(A)** The SRAMP database predicted the existence of multiple potential theoretical binding sites for ANGPTL3 m^6^A modification. **(B, C)** Expression of ANGPTL3 precursor and mature mRNAs in METTL3-silenced AGS and HGC27 cells was verified using qRT-PCR. **(D, E)** Half-life of intracellular ANGPTL3 mRNA was prolonged after METTL3 silencing. **P < 0.01, ***P < 0.001

Previous studies have demonstrated that METTL3 participated in alterations of multiple biological functions in various cancer types by an m^6^A-independent or dependent manner [23, 24]. Based on the obtained data and also to further confirm that METTL3 was dependent on its m6A methyltransferase activity in STAD to weaken ANGPTL3 expression, we first predicted the possible m^6^A modification sites in ANGPTL3 sequences in the publicly available online database SRAMP. And each possible m^6^A site was evaluated by virtue of the assigned prediction score. As shown in Fig. 5A, there were 14 possible m^6^A sites in the ANGPTL3 sequence, of which one had very high confidence and four had high confidence. The effect of mRNA methylation on mRNA fate was controlled by the position of the methylation site or other factors associated with the mRNA. Next, we analyzed the expression of precursor (pre) and mature (mat) mRNAs of ANGPTL3 in si-METTL3 group and si-NC group cells. We found that the precursor mRNA of ANGPTL3 does not change in response to a decrease in METTL3 levels relative to a significant increase in mature mRNA (Fig. 5B C). Similarly, effective prolongation of the half-life of intracellular ANGPTL3 mRNA was detected in AGS and HGC27 cells after si-METTL3 transfection and Actinomycin D treatment (Fig. 5D and E). Therefore, the attenuated METTL3-mediated m^6^A modification might be the reason for the increased stability of ANGPTL3 mRNA in si-METTL3 STAD cells.

### METTL3 silencing impedes tumor growth in vivo

**Fig. 6:**
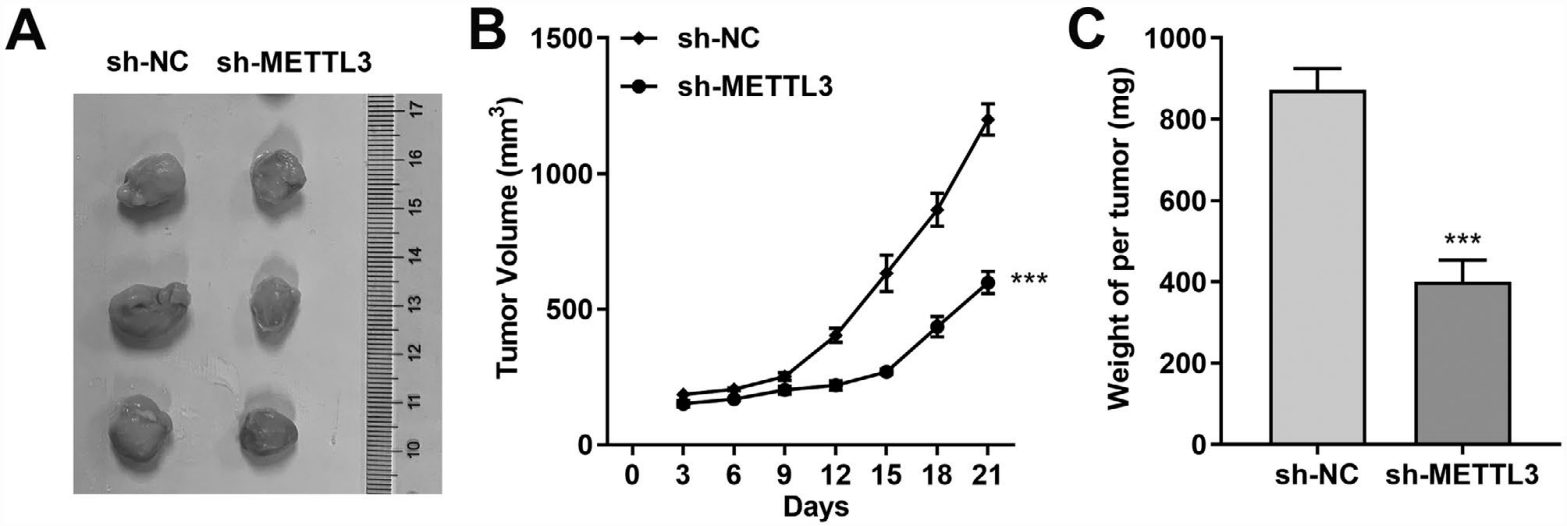
In vivo METTL3 silencing inhibited AGS cell-dominated tumor formation. **(A)** Representative images clearly show xenograft tumors in nude mice injected subcutaneously with AGS cells. **(B)** Tumor volumes in xenograft mice were monitored regularly (three days/time). **(C)** METTL3-knockdown AGS cells were significantly diminished xenograft weight 21 days after subcutaneous injection into nude mice. ***P < 0.001

We continued to focus on the contribution of METTL3 to stomach adenocarcinoma cell growth in vivo. We stably knocked down the expression of METTL3 in AGS cells with the help of shRNA targeting METTL3, and subsequently injected sh-NC AGS cells and sh-METTL3 AGS cells subcutaneously into nude mice. After three weeks, it was clearly observed that the tumor volume formed by sh-NC AGS cells was obviously larger than that from sh-METTL3 AGS cells (Fig. 6A and B). Besides, the tumors in the sh-NC group weighed more than those in the sh-METTL3 group (Fig. 6C). These findings illustrated that the reduction of METTL3 was responsible for the slowed tumor growth in vivo.

## Discussion

Since the 1970s, m^6^A has been recognized as one of the most prevalent and plentiful internal modifications of mRNA in mammals [25–27]. Typically, this RNA chemical labeling installed by m^6^A “writer” proteins is dynamically reversible and goes beyond simple mRNA modifications, and is considered essential in regulating physiological processes in a variety of diseases [28]. Real data revealed that, as the catalytic subunit of the m^6^A methyltransferase complex or “writer”, METTL3 relies on its methyltransferase activity to shape biological behavior in very different ways under normal and diseased conditions [29], including the regulation of tumor cell proliferation, metastasis, and invasion [30]. Notably, the regulation of m^6^A by METTL3 appears to have conflicting results (inhibition or promotion) in the induction of cancer progression. One study noted that high METTL3 expression impeded glioma growth in vitro and in vivo, mainly attributed to the maintenance of intracellular mRNA m^6^A levels by METTL3 [31]. In some cancers, intracellular m^6^A hypomethylation in tumor cells due to decreased METTL3 expression was also found, which in turn induced recurrence of ocular melanoma and endometrial tumors [32, 33]. In contrast, in gastrointestinal tumors, METTL3 is mainly responsible for oncogenic effects. For example, METTL3 is a key promoter of gastric cancer metastasis in vivo and malignant progression in vitro, activating downstream regulatory networks through regulation of m^6^A-dependent ZMYM1 [17]. These paradoxical phenomena are mainly attributed to the high cancer heterogeneity and differences in targeting pathways.

In the present study, we reported that METTL3 inhibited ANGPTL3 in an m^6^A-dependent manner. Consistent with previous reports [34], We confirmed the high expression of METTL3 in 18 pan-cancer types including STAD from the TCGA database. Clinicopathological analysis showed that high METTL3 level was associated with STAD stage and distant metastasis. Second, functionally, METTL3 silencing impeded STAD cells growth and motility and retarded the growth of tumors induced by AGS cells in vivo. Considering the controversial findings of METTL3 in cancer, the results we obtained fully demonstrated and revealed the oncogenic role of METTL3 in promoting STAD progression. Participants in the m^6^A methylation machinery are related to STAD, but it is uncertain whether they depend on the altered m^6^A modification. And another important result of this study is to determine the causal connections among ANGPTL3 mRNA m^6^A levels and METTL3 expression and STAD tumor progression using bioinformatics analysis and comprehensive experiments.

METTL3 can activate cell proliferation by installing m^6^A on key genes that are critical for blocking cell growth [35]. In this study, we observed that METTL3 recognized the m^6^A sites in ANGPTL3 mRNA and negatively modulated the stability of ANGPTL3 mRNA. This conclusion was verified by MeRIP-PCR. More than that, we tested a significant negative correlation between METTL3 and ANGPTL3 in 62 pairs of gastric cancer tissues. In fact, we cannot exclude that METTL3 may also depend on other m^6^A regulators to exert m^6^A modifying effects on ANGPTL3, which still needs further investigation. As a member of the angiopoietin-like protein family, ANGPTL3 can be involved in several disease biological processes including tumor angiogenesis, cancer cell migration and proliferation, and tumor thrombus formation [36]. It has been shown that ANGPTL3 exhibited potential oncogenic effects in oral cancer [37], hepatocellular carcinoma [38], as evidenced by the high intracellular expression of ANGPTL3 that successfully increased the tumorigenicity and invasiveness of the target cells. Similarly, a recent study reported that ANGPTL3 depletion inhibited cervical cancer cells viability [39]. Nevertheless, the role of ANGPTL3 in cancer progression was rely on the certain tumor type, where it has also been illustrated that ANGPTL3 assumed the role of tumor suppressor in gastric cancer [40]. Here, we performed additional in vitro studies on the effects of ANGPTL3 on the biological behaviors of gastric cancer cells. In AGS and HGC27 cell lines, ANGPTL3 expression was significantly hindered under METTL3 and markedly strengthened under si-METTL3, while cell growth and migration changes were opposite to ANGPTL3 levels but consistent with ANGPTL3 mRNA m^6^A levels, suggesting that ANGPTL3 antagonized STAD tumors. Based on these findings, we provided some new insights into METTL3-mediated regulatory networks. M6A modulators have attracted increasing attention as therapeutic targets. Although studies on the oncogenic role of METTL3 in human cancers are still limited, potential clinical applications for METTL3 have recently emerged in the form of substrate competitive inhibitors of METTL3 [41]. SAM mimics were found to be the first inhibitors of METTL3 [41]. However, it was unknown whether this compound could reduce substrate RNA methylation responses as well as substrate m6A levels due to cell penetration issues and non-specific targeting. Therefore, continued exploration of METTL3 or association of METTL3 with “specific sites” in ANGPTL3 mRNA might contribute to the development of new cancer therapies and lead to the development of a new pathway for effective METTL3 inhibition in gastric cancer. Importantly, it is feasible to treat cancer and other diseases by using METTL3 inhibitors in combination with other therapies.

## Conclusion

In the current study, we systematically analyzed the levels of METTL3 and its co-expressed gene ANGPTL3 in STAD patients and their correlation. Our results suggested that aberrant METTL3 expression was essential for the malignant progression of STAD, and its presence contributed to tumor cells growth and metastasis. More than that, METTL3 levels were negatively correlated with ANGPTL3, because recruitment of METTL3 increased m^6^A modification-induced ANGPTL3 mRNA degradation. Overall, ANGPTL3 may be a useful biomarker in gastric cancer, and the combination of “writer” METTL3 and ANGPTL3 exhibits an innovative m^6^A-dependent regulatory mechanism.

## Electronic supplementary material

Below is the link to the electronic supplementary material.

Supplementary Material 1

Supplementary Material 2

## Data Availability

All data generated or analyzed during this study are included in this published article.

## Abbreviations

m^6^A: N6-methyladenosine
METTL3: Methyltransferase-like 3
STAD: Stomach adenocarcinoma
ANGPTL3: Angiopoietin-like 3
GC: Gastric cancer
mRNAs: Messenger RNAs
OD: Optical density
ANOVA: Analysis of variance.
