# Supplementary material for: METTL3 regulates N6-methyladenosine modification of ANGPTL3 mRNA and potentiates malignant progression of stomach adenocarcinoma
Source: BMC Gastroenterol. 2023 Jun 21;23:217. doi: 10.1186/s12876-023-02844-x (PMC10283274; doi:10.1186/s12876-023-02844-x)
Supplement: Supplementary file 1 — Supplementary Material 1 [file 12876_2023_2844_MOESM1_ESM.docx]

**Additional file 1**

**Results**


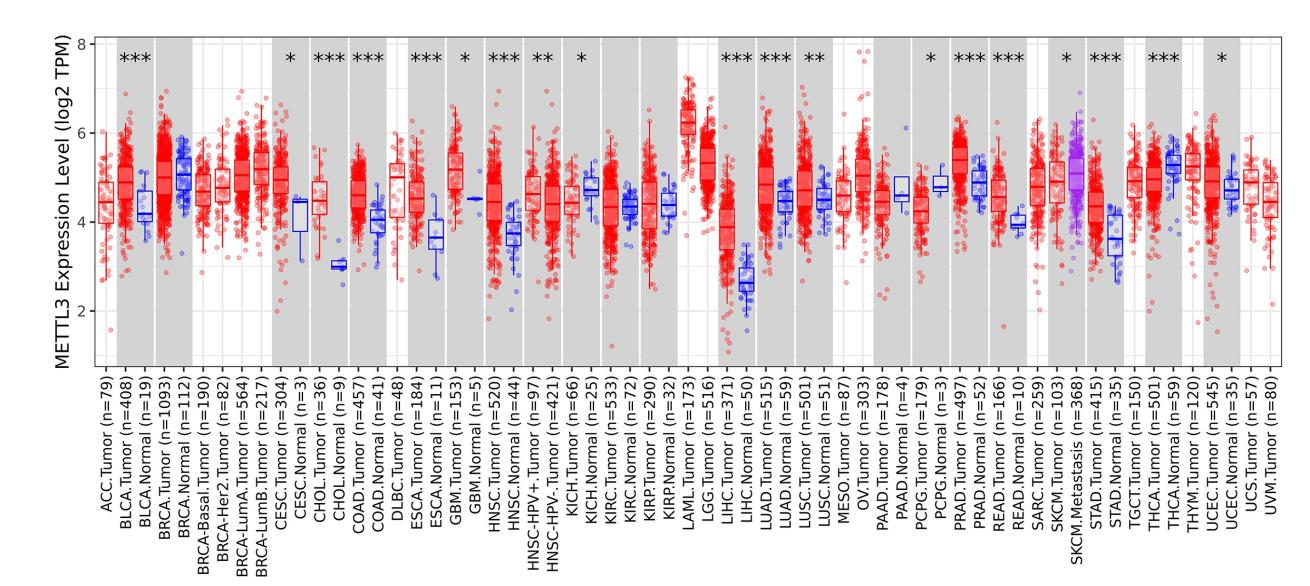


**Figure. S1 METTL3 was present in a high expression pattern in various cancers.** Box plots were conducted to represent the differential expression of METTL3 in 33 cancer types (ACC, BLCA, BRCA, CESC, CHOL, COAD, DLBC, ESCA, GBM, HNSC, KICH, KIRC, KIRP, LAML, LGG, LIHC, LUAD, LUSC, MESO, OV, PAAD, PCPG, PRAD, READ, SARC, SKCM, STAD, TGCT, THCA, THYM, UCEC, UCS, UVM) . The blue and red box lines indicated normal and tumor tissues, respectively. Differences between clinical tumor samples and paired TCGA normal controls were assessed using Student's *t*-test and represented using box plots. We obtained METTL3 expression in 33 cancers from the publicly available TCGA database. **P*< 0.05, ***P*< 0.01, ****P*< 0.001.


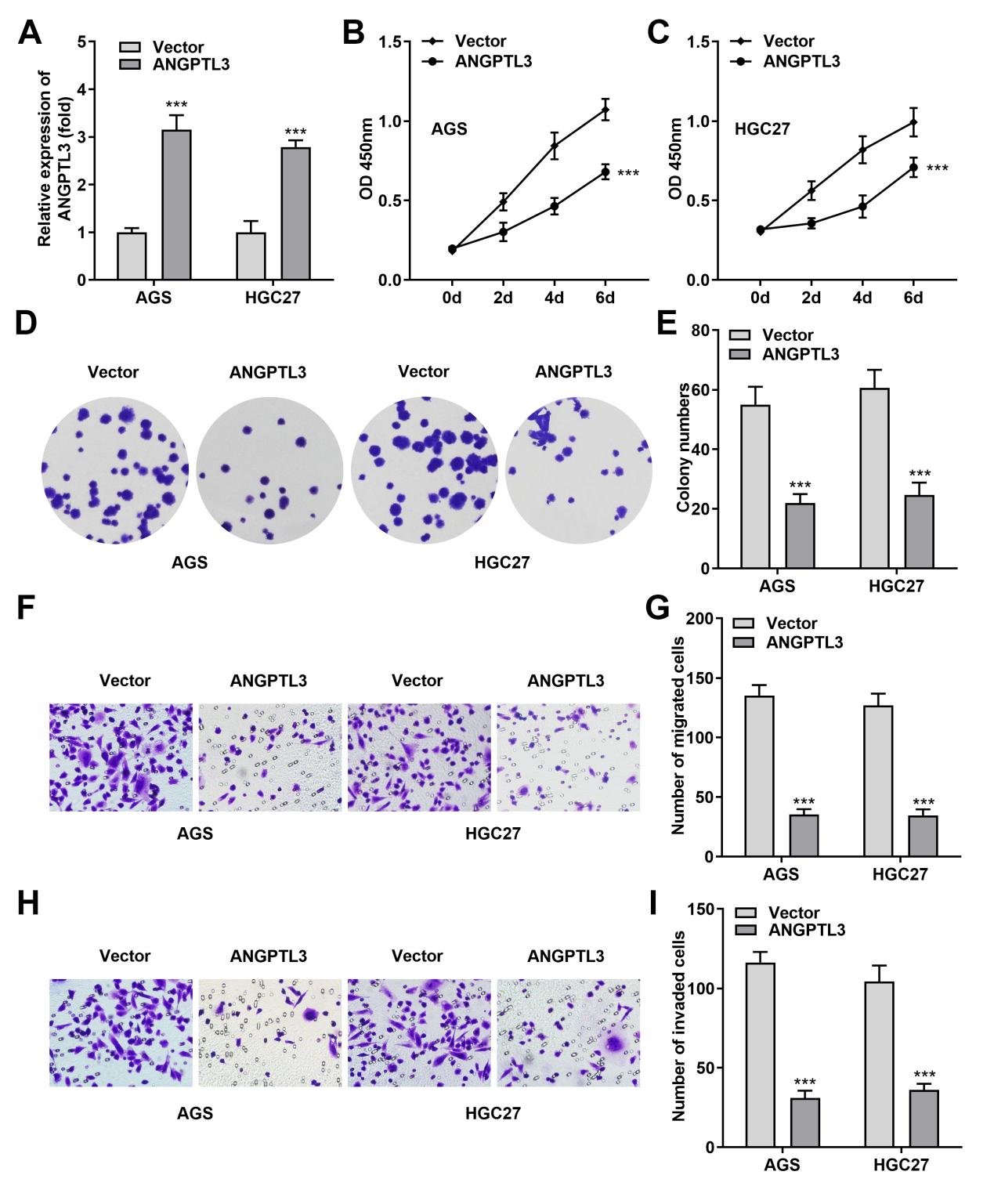


**Figure.S2 ANGPTL3 overexpression impeded STAD cell proliferation, migration and invasion.** (A) RT-qPCR was used to verify the expression of ANGPTL3 in AGS and HGC27 cells. (B, C) CCK-8 assay to obtain cell viability change data. (D, E) Colony formation assay was possessed to obtain changes in cell proliferation capacity. The number of AGS and HGC27 cell clones was assessed by the obtained microscopic images. (F-I) Transwell assay illustrated that ANGPTL3 over-expression hindered the migration and invasion of AGS and HGC27 cells. The number of migrating and invading AGS and HGC27 cells was calculated based on the obtained images. ****P*<0.001.
