# Supplementary material for: METTL3 regulates N6-methyladenosine modification of ANGPTL3 mRNA and potentiates malignant progression of stomach adenocarcinoma
Source: BMC Gastroenterol. 2023 Jun 21;23:217. doi: 10.1186/s12876-023-02844-x (PMC10283274; doi:10.1186/s12876-023-02844-x)
Supplement: Supplementary file 2 — Supplementary Material 2 [file 12876_2023_2844_MOESM2_ESM.docx]

**
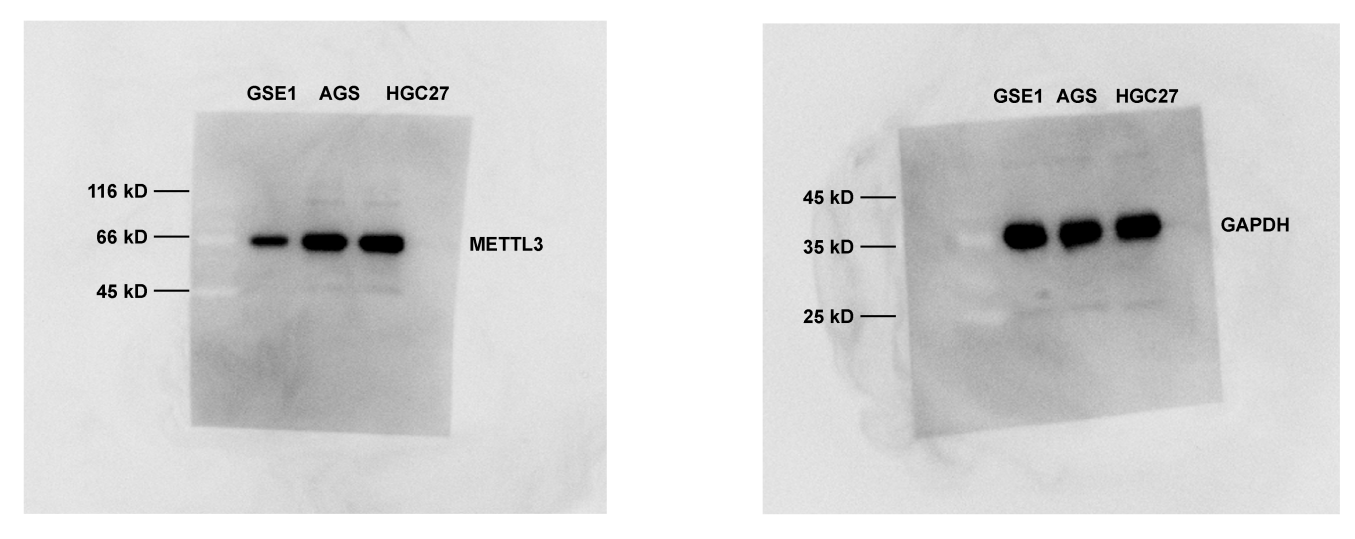
**

**Figure S3: Uncropped and unedited versions of the blots in Figure 1C.**


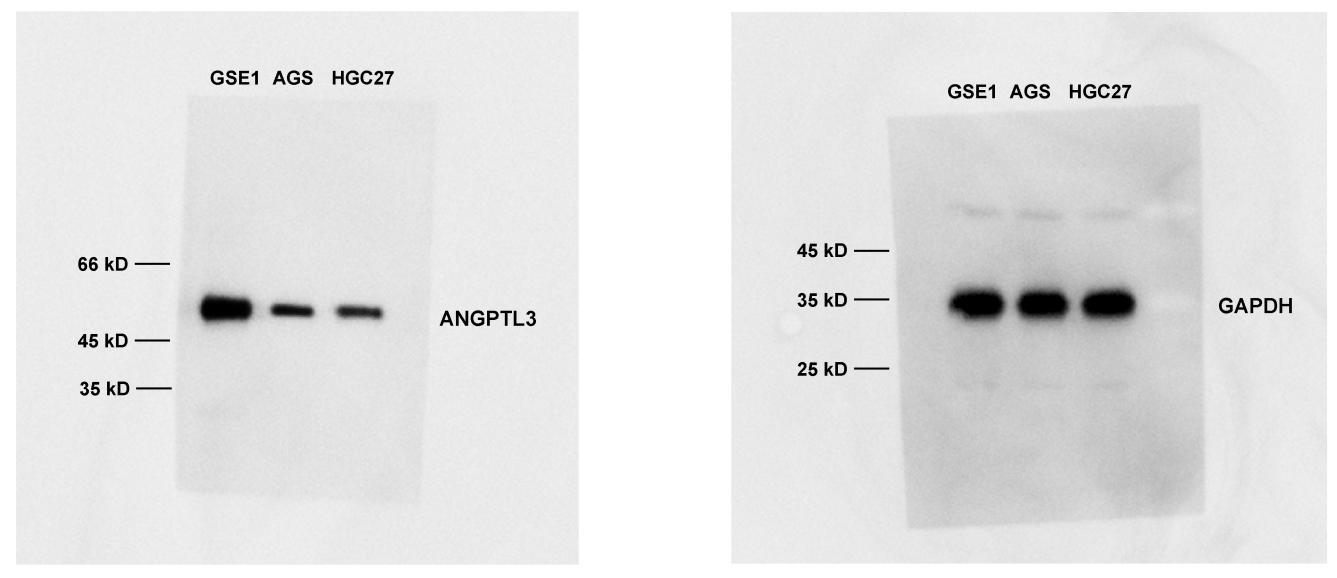


**Figure S4: Uncropped and unedited versions of the blots in Figure 3D.**


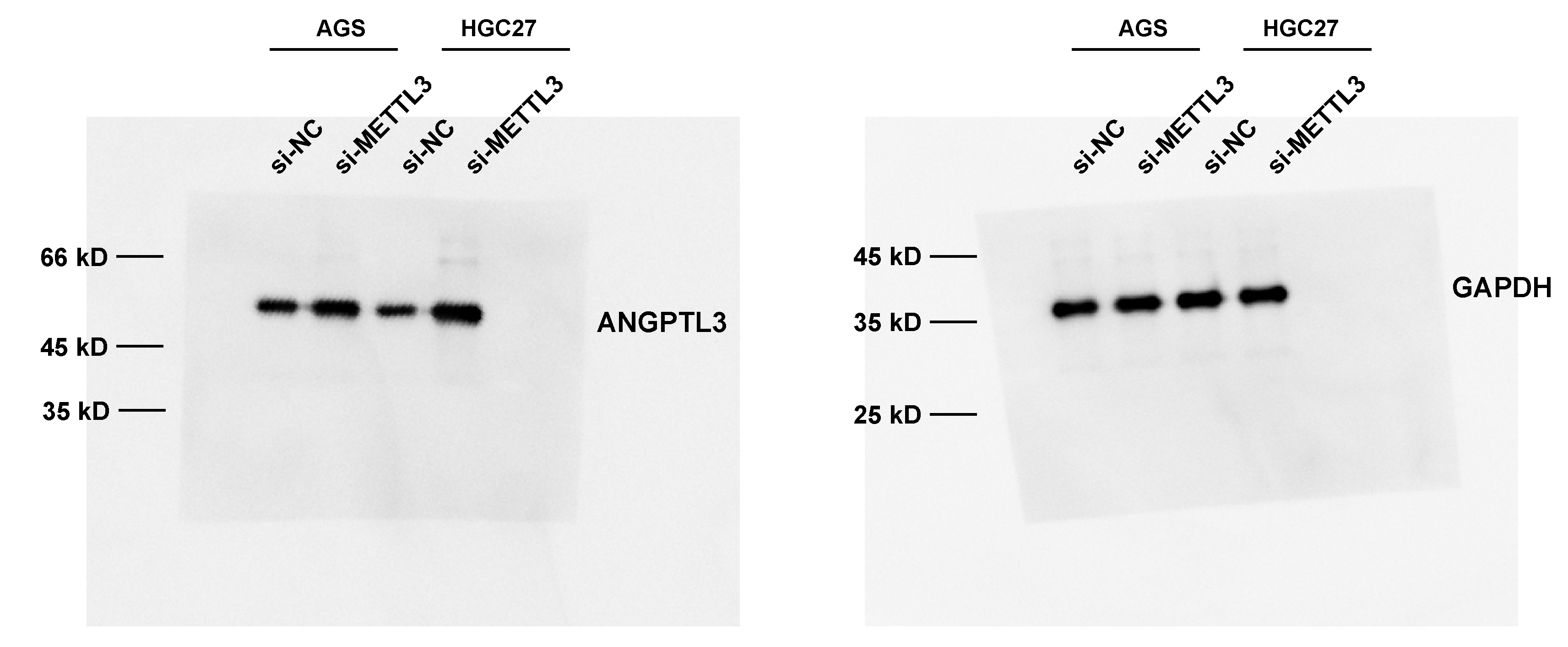


**Figure S5: Uncropped and unedited versions of the blots in Figure 3G.**

**To conserve antibodies, the membrane for Western blotting was cropped according to the molecular weight of the protein prior to incubation with primary antibodies.**
